# Physical Properties and Durability of Lime-Cement Mortars Prepared with Water Containing Micro-Nano Bubbles of Various Gases

**DOI:** 10.3390/ma14081902

**Published:** 2021-04-11

**Authors:** Małgorzata Grzegorczyk-Frańczak, Danuta Barnat-Hunek, Wojciech Andrzejuk, Jacek Zaburko, Monika Zalewska, Grzegorz Łagód

**Affiliations:** 1 Faculty of Civil Engineering and Architecture, Lublin University of Technology, Nadbystrzycka St. 40, 20-618 Lublin, Poland; m.grzegorczyk@pollub.pl; 2 Faculty of Technical Science, Pope John Paul II State School of Higher Education in Biała Podlaska, Sidorska St. 95/97, 21-500 Biała Podlaska, Poland; w.andrzejuk@dydaktyka.pswbp.pl (W.A.); monika.zalewska.budownictwo@gmail.com (M.Z.); 3 Faculty of Environmental Engineering, Lublin University of Technology, Nadbystrzycka St. 40B, 20-618 Lublin, Poland; j.zaburko@pollub.pl

**Keywords:** micro-nano bubble water, O_2_, O_3_, CO_2_, lime-cement mortars, nano admixtures

## Abstract

The paper presents the experimental studies on the effect of the water containing micro-nano bubbles of various gases on the physico-mechanical properties of lime-cement mortars. In total, 7 types of mortars were prepared: with water containing the micro-nano bubbles of O_2_, O_3_ or CO_2_ as 50% or 100% substitute of ordinary mixing water (tap water) and the reference mortar prepared using tap water. In order to determine the influence of water with micro-nano bubbles of gases, the consistency of fresh mortar and the physical properties of hardened mortar, i.e., specific and apparent density, total porosity, water absorption by weight and capillary absorption, were established. The mechanical strength of the considered mortars was studied as well by conducting the tests for flexural and compressive strengths following 14, 28 and 56 days. Reduced workability and capillary absorption were observed in the modified mortars within the range of 0.9–8.5%. The mortars indicated an increase in the flexural strength after 28 days ranging from 3.4% to 23.5% and improved compressive strength in 1.2–31%, in comparison to the reference mortar. The conducted studies indicated increased flexural and compressive strengths along with the share of micro-nano bubbles of gases in the mixing water.

## 1. Introduction

Nanotechnology is a branch of science and technology concerned with production, study and application of nanomaterials, i.e., particles with the size under 100 nm in at least a single dimension [1]. The nanoscale materials may acquire new, different structural, chemical, optical, thermal, and mechanical properties in comparison with the macroscale materials [2]. This is due to the large specific surface of nanomaterials, which in turn may lead to higher chemical reactivity [3]. When the size of nanosubstance fragments is being reduced, no changes in the physicochemical properties are noted in the initial stage. Then, it begins changing at some point, first linearly and then in a strongly non-linear manner. The materials with the size below 50 nm are subject not only to the laws of both classic physics, but predominantly to the laws of quantum physics [4], having different structural, chemical, optical, thermal, and mechanical properties from the larger scale materials. Due to the unique properties of the produced materials, nanotechnology is widely employed in various branches of industry, cosmetology, pharmacy, medicine, environmental engineering, agriculture or materials engineering [3,4,5,6]. Micro-nano bubbles in water may constitute an example; in this case, surface tension appears inside micro-nano bubbles under the influence of high pressure, which greatly increases their density [7,8].

Civil engineering is also a sector in which the nanomaterial-related solutions from other branches of science are frequently adapted, as well as produced. In the recent years, a phenomenon consisting in the development of nanomodification of popular construction materials, i.e., steel, concrete and timber, can be observed. Selected nanoadditives can grant better physical, mechanical and strength properties to mortars and concretes [1,2,3]. The nanosilver addition confers the bactericidal and fungicidal properties to concretes [5,9]. In turn, nano SiO_2_ increases the pozzolanic reactivity of a concrete mixture, improves the compressive strength, as well as decreases porosity and water absorptivity [10,11]. The C-S-H nanoparticles accelerate the increase of concrete early strength [12]. The concrete with nano TiO_2_ acquires the self-cleaning properties [13]. Nano aluminum oxide improves the concrete microstructure, increases the compressive strength and reduces the binding time [14]. Introduction of graphene oxide to the cement slurry increases the hydration degree and reduces the structure of pores, contributing to improved compressive and tensile strength, even up to 40% [15]. The latest research conducted by the authors [16] indicated that nanocrystalline cellulose used as a surface hydrophobizing agent may efficiently reduce the porosity of cement mortars, as well as improve the compressive and tensile strengths.

Micro-nano bubble (MNB) water is a type of nanomaterial. It is created via suspending the gas bubble in liquid, and owing to its unique properties and availability, it is employed in numerous branches of industry, which quickly implemented this beneficial solution [7,8]. The micro-nano gas bubble water found wide application in agriculture, cosmetology, medicine, and environmental engineering. Due to its physicochemical properties, i.e., long water retention time, high gas pressure inside the bubble and stability, it is used in numerous applications.

Micro-nano bubble technology found application for water purification as well as wastewater treatment and may become a good alternative solution for the global environmental problems connected with degradation and depletion of water resources [17]. The water treatment process with the use of nanotechnology constitutes a chance to restore the natural ecosystem [17,18]. The use of micro-nano bubble water for efficient removal of microbiological pollution from various surfaces [8] may be an alternative for the chemicals used in cleaning agents which cause irritation of skin or the respiratory system. Moreover, it has a beneficial effect on the growth of organisms, as well as find wider application in the treatment of wastewater [19,20]. Studies also prove that the oxygen micro-nano bubble water accelerates the growth of plants, increases blooming, has a bactericidal effect and improves the soil structure [21]. In turn, the ozone micro-nano bubble water has bactericidal and preserving effect; it is used in wastewater treatment, as well as in medicine and stomatology [22]. The water with carbon dioxide is used in treating skin problems, inflammations, and plant nourishment [23]. The nitrogen micro-nano bubble water is applied in plant fertilization [24]. In turn, the water with hydrogen is widely applied in medicine, dietetics, and biotechnology [17,24].

In recent times, attempts have been made to find the application for micro-nano bubble water in the technology of modifying cement composites. It is known that the concrete prepared using micro-nano air bubbles (MNAB) water is characterized by greater workability [17], increased heat of hydration [25] and accelerated binding time [26]. Kim et al. [27] observed 31% increase in the compressive strength of mortars prepared using Portland cement and hydrogen micro-nano bubble water as mixing water. The studies performed by Asadollahfardi et al. [28] indicated that the compressive strength of concrete increased by 13.2%, and the tensile strength improved by 16.4, in relation with the control sample, following substitution of ordinary mixing water with MNAB water. Moreover, an increase in water absorption by weigh by as much as 67% was noticed. Mohsen Zadeh et al. [29] indicated the influence of MNAB water on the reduction of capillary absorption of the investigated concretes by 16% and 20%, depending on the amount of water exchange. Kim et al. [30] stated that the properties of polymer-cement mortars for repairing concrete structures can be effectively improved through simultaneous application of calcium nitrate with the CO_2_ micro-nano bubble water.

Unfortunately, during the review of the available literature, no studies on the application of various gases in micro-nano bubble water or the difference in their effect on the properties of concretes were found. The results of afore-mentioned studies mainly pertain to the application of mixing water with air and water saturated with CO_2_. No publications in which admixtures of other gases were added, are available. No papers on the MNB water modification of lime or lime-cement mortars were identified either. Therefore, the lack of the afore-mentioned data was determined as a gap in knowledge to be bridged during the studies conducted in this work.

## 2. Materials and Methods

### 2.1. Material Properties

The paper focused on determining the influence of the ozone, oxygen, and carbon dioxide micro-nano bubble water on the physico-mechanical properties of lime-cement mortars. This type of mortars was chosen, because they can be successfully used as renovation mortars for the reconstruction of stone walls (especially limestone) and conservation of historical buildings and constructions [31]. Lime, although characterized by lower strength than cement, confers beneficial properties to mortars, i.e., surface adhesion and elasticity [32]. Moreover, the mortars with high lime content are characterized by high water vapor permeability, which enables to transport moisture outside the wall. Lime also limits the possibility of efflorescence on walls, and mitigates the biological corrosion processes (including algae, fungi, and bacteria) [33].

A reference mortar (REF) was prepared using tap water in order to conduct a comparative analysis. Six batches of mortars with the water containing micro-nano bubbles of above-mentioned gases were prepared, substituting the ordinary mixing water (tap water) in 50% and 100%. The consistency of a fresh mixture, as well as the basic physical properties were examined. In order to determine the mechanical properties, the compressive and flexural strength tests were performed after 14, 28, and 56 days.

The research involved preparing seven types of lime-cement mortar:

- Reference—reference mortar with ordinary tap water;
- 50% CO_2_—mortar with 50% CO_2_ micro-nano bubble water;
- 100% CO_2_—mortar with 100% CO_2_ micro-nano bubble water;
- 50% O_2_—mortar with 50% O_2_ micro-nano bubble water;
- 100% O_2_—mortar with 100% O_2_ micro-nano bubble water;
- 50% O_3_—mortar with 50% O_3_ micro-nano bubble water;
- 100% O_3_—mortar with 100% O_3_ micro-nano bubble water.

The composition of mortars was given in Table 1.

The weight ratios of particular mortar ingredients was established as follows: water:binder:sand ↔ 3:4:3. The w/b (water/binder) ratio amounted to 0.75.

Hydrated lime, with the bulk density of 0.51 kg/dm^3^ and specific density of 2.24 kg/dm^3^ (obtained from ALPOL, Chemical and Mineral Raw Materials Plant “Piotrowice”, Poland), constituted the main binder in the analyzed mortars. In order to improve the strength, the second binder was applied, i.e., white Portland cement CEM I 52.5 R (AALBORG Portland, Aalborg, Denmark) with bulk density of 1.22 kg/dm^3^ and specific density of 3.13 kg/dm^3^. The weight ratio of cement to hydrated lime amounted to 1:3. The binder parameters, conforming to PN-EN 459-1:2015-06 [34] and PN-EN 197-1:2012 [35] were presented in Table 2 and Table 3.

Natural quartz sand (0.0–2.0 mm fraction) with bulk density of ρ_p_ = 2.70 kg/dm^3^ constituted the fine aggregate used in mortars. It was characterized by the following chemical composition by weight: 95.3% SiO_2_, 1.9% Al_2_O_3_, 0.7% Fe_2_O_3_, 0.40% CaO, 1.7% others [38].

Oxygen, ozone or carbon dioxide micro-nano bubble water was used as a partial or complete substitute of tap water.

### 2.2. Methods of Micro-Nano Bubbles of Gases Obtaining

Dispersion of the gas in water was carried out using the rotating pump technology and ceramic nanoporous membranes. Such setup enabled to achieve the typical diameter of nanobubbles ranging from 80 to >200 nm. Their size and amount, were regulated using several physical parameters, such as pressure, as well as liquid and gas flow rate. The higher the liquid flow rate, the greater the shear stresses and the lower the gas volume which can be detached from the membrane surface [7,39,40,41].

**Figure 1:**
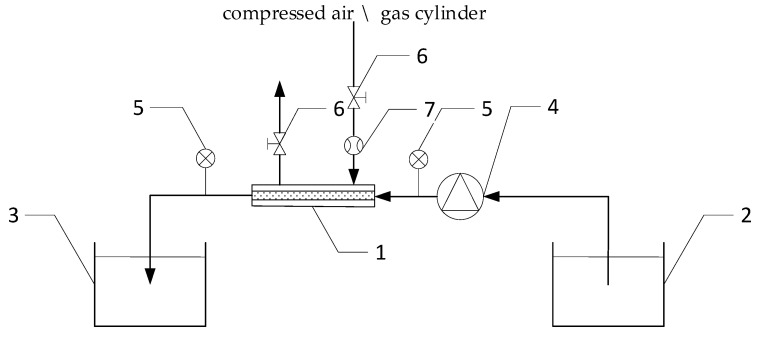
Scheme of the technological installation for the micro-nano bubble water generation: 1—micro-nano bubble generator with a ceramic membrane, 2—primary tank, 3—micro-nano bubble mixing water tank, 4—pump, 5—manometer, 6—valve, 7—rotameter.

Figure 1 presents the scheme of the technological system for the generation of micro-nano bubbles in the mixing water, in which a tubular ceramic membrane with SiO_2_ was employed as the generator of micro-nano bubbles.

The mixing/tap water from the primary tank (2) is pumped using a pump (4) to the micro-nano bubble generator with a ceramic membrane (1). The micro-nano bubble generator is supplied with compressed gas (from a tank). Having passed through the generator, the micro-nano bubble water is directed via a pipe to the tank (3). The installation is equipped with a measurement system, comprising manometers (5) and a rotameter (7).

The system of prime importance for the micro-nano bubble production is the micro-nano bubble generator. It comprises a tubular membrane made of nanoporous material, with channels having irregular shape. The mixture of water and gas flows through these channels. The membrane is placed in a stainless steel housing. The compressed air or gas supplied to the generator under pressure passes through the nanopores of the membrane. The micro-nano bubble generation involves two main phases: their growth (bubbles expansion) and their detachment from the membrane. During the process of micro-nano bubble generation, the bubble formed by the membrane surface pores is affected by a number of forces. Surface tension acts upon the micro-nano bubble as the force keeping the bubble inside the nanopore. The resistive force exerted by the flow of the aqueous phase acts upon the micro-nano bubble as a shear force, detaching the bubble from the nano pores. The detached micro-nano bubble initially has the same diameter as the pores of the membrane material and forms a half-sphere, whereas the gas pressure inside the bubble is at maximum. Then, due to the micro-nano bubble expansion, the gas pressure inside drops. The resistive force caused by the flow of the aqueous phase increases along with the diameter of bubbles. When the shear force becomes greater than the force holding the micro-nano bubble at the membrane surface, it detaches from the nanopore. This results in the generation of monodisperse micro-nano bubbles with the diameter slightly greater than that of pores (the diameter of pores ranges from 50 to 150 nm).

Micro-nano bubble generation using ceramic membranes constitutes one of the numerous available methods. The micro-nano bubbles can also be generated with the vortex flow method, where the gas and liquid are forced into vortex motion at high speed, and micro-nano bubbles are generated by the shear force. Fan et al. managed to generate the micro-nano bubbles with the mean diameter lower than 50 nm using a venturi [41] and a gas introduced to the pipe. Ahmadi et al. [42] generated nanoparticles with the mean diameter of 130–545 nm using a venturi and the hydrodynamic cavitation phenomenon. In turn, Wu et al. [43] generated the micro-nano bubbles with the diameter of <500 nm by applying cavitation induced as a result of high mixing intensity of the liquid. Acoustic cavitation is another method. An acoustic probe generates the sound waves with the frequency of 20 kHz, producing vibrations and transferring it onto the gas nozzle submerged into a liquid. This method was employed by Oeffinger and Wheatley, who generated the micro-nano bubbles with the mean diameter of 400–700 nm, by introducing a stream of gas into the solution under a regular pressure [44]. The literature review confirms the fact that the frequencies under 20 kHz do not destroy micro-nano bubbles, and even enable generating additional micro-nano bubbles through the cavitation of gases dissolved in the liquid [45]. The application of simple, scalable methods of micro-nano bubble generation with low energy consumption is of key importance for their prospective applications in the future. This knowledge was supported by the tests carried out by independent laboratories using the Nano Sight instrument by Malvern [46,47,48].

### 2.3. Methods of Mortar Analysis

The research schedule involved conducting the following determinations: bulk density, total porosity, water absorption by weight, water absorption coefficient as well as mechanical properties of lime-cement mortars.

The consistency of fresh mix was determined through the flow table test, in line with the PN-EN 1015-3:2000 standard [49]. The mean from two perpendicular measurements of sample flow diameter was assumed as the result.

Bulk density was determined by applying the hydrostatic method, in accordance with the PN-EN 1015-10:2001 standard [50], which involves weighing the material saturated with water in air and then under water and calculating the volume from the mass difference. Specific density was determined with the pycnometric method, in line with PN-EN 1936:2010 [51]. Prior to the investigation, the samples were dried to constant mass at a temperature of (105 ± 5) °C, then the material was ground in an electric ball mill. The ground material in the amount of 10 g was used in each pycnometer.

Total porosity, determining the share of pore volume in the total volume of material, was calculated in line with [51], using the formula below:

(1) $$P=\left(1\;-\;\frac{\rho_{b}}{\rho_{r}}\right)\times 100\%$$

$\rho_{b}$—bulk density [g/cm^3^],
$\rho_{r}$—specific density [g/cm^3^].

Water absorption by weight, which constitutes the capacity for water absorption of the material at atmospheric pressure, was determined in accordance with PN-EN 13755:2008 [52] as the ratio of the weight of absorbed water to the weight of dry sample. The samples were saturated until no change in weight was noted. Then, the samples were dried to constant weight at the temperature of (105 ± 5) °C.

Capillary rise was studied in line with the PN-EN 1015-18:2003 standard [53]. Six samples were prepared for each series of investigated mortars. Their weight was determined after 10 min, 90 min, and 24 h of water rise by a single surface of the sample (surface with the dimensions of 40 × 40 mm). According to the standard, the water absorption coefficient constitutes the measure of capillary rise. It was calculated using the following formula:

(2) $$C=0.625\left(m_{w24}\;-\;m_{s}\right)$$

where:

*C*—water absorption coefficient [kg/m^2^],
*m_w_*_24_—sample weight determined after 24 h of capillary water rise [g],
*m_s_*—sample weight after drying to constant mass [g].

**Figure 2:**
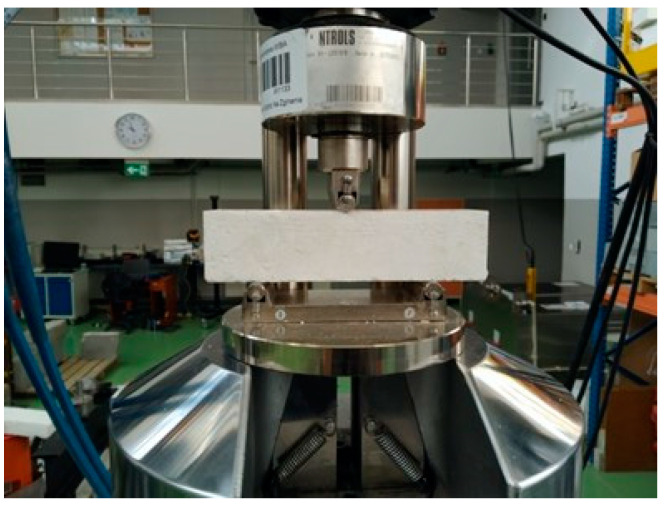
Flexural strength test conducted on the 50% CO_2_ sample after 28 days.

**Figure 3:**
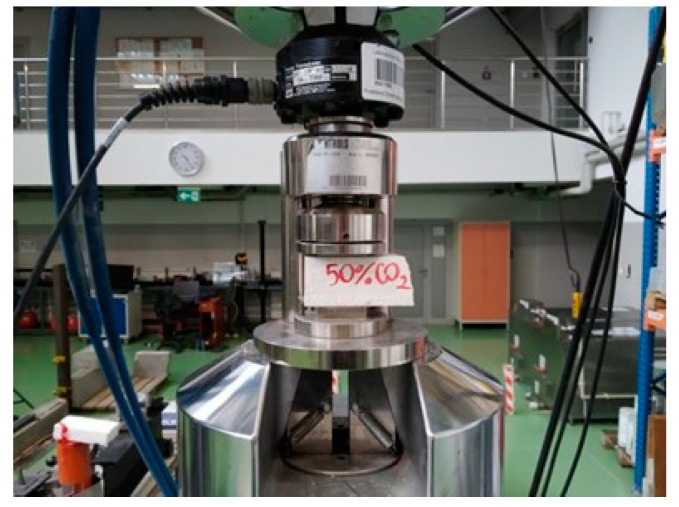
Compressive strength test conducted on the 50% CO_2_ sample after 28 days.

The studies aimed at determining the mechanical properties of the analyzed lime-cement mortars involved in the flexural and compressive strength tests. The tests were performed in accordance to the PN-EN 1015-11:2001 standard [54] using bar samples with the dimensions of 40 × 40 × 160 mm. The flexural strength tests were conducted first, so that the broken parts could be subsequently used in the compressive strength test. The strength tests (Figure 2 and Figure 3) were performed after 14, 28 and 56 days.

### 2.4. Visualization of Obtained Data Using Multidimensional Scaling Method

The multidimensional scaling (MDS) method was used for visualization of the similarity level for the component of matrix containing the data connected with the properties of the examined mortars along with the different amount (50%, 100%) of used MNW with CO_2_, O_2_ and O_3_. The applied MDS method [55,56,57] allows obtaining sets of points distributed in a two-dimensional space in such a way that in the analyzed cases (tested mortars—with dose of MNW and reference), the similar points were closest to each other, while different ones were distributed far apart [58]. Its aim was to show the overall similarity between the entire analyzed set of mortar samples in one figure, simultaneously taking into account all the analyzed properties (measured parameters) of lime-cement mortars changing due to the substitution of tap water with micro-nano bubble water. Scaling is performed at the beginning with certain initial configuration. Then, the points are shifted iteratively, as a result of which the match between the distances and data is improved until subsequent iterations bring no further improvement [59]. Generally, it can be stated that the more accurately the data correspond to the distances in MDS, the better the MDS configuration represents the proximity structure (e.g., sample measurement set with various modifications). If the match of MDS is good (e.g., measured by means of STRESS—Standardized REsidual Sum of Squares), they can be checked and interpreted visually, which was done in this work.

## 3. Results and Discussion

Table 4 presents the consistency of fresh mortars measured with the flow table method.

The consistency tests of lime-cement mortars prove the effect of micro-nano bubble water on the workability of prepared mixes. The use of micro-nano bubble water instead of the mixing water resulted in a reduction of mortar fluidity and workability. The greatest drop in mix fluidity was observed in the mortars with the CO_2_ micro-nano bubble water. This is connected with the accelerated binder hardening processes. Hydrated lime, forming an air-hardened binder after being mixed with water, hardens as a result of a slow carbonatation process, reacting with the carbon dioxide from the atmosphere and forming calcium carbonate. Application of the CO_2_ micro-nano bubble water, instead of ordinary mixing water, accelerated the hardening process.

MNB water also affects the cement mixes. Khoshroo et al. [25] investigated the effect of MNAB water on concrete properties. It turned out that micro-nano bubble water reduces the workability of fresh concrete. However, as the amount of mixing water substitution with micro-nano bubble water increases, so does workability in relation to the control sample. When 30% micro-nano air bubble water was applied, workability deceased by 12%; in turn, increasing the amount of MNAB water reduced workability by 7%.

Arefi et al. [26] investigated the concrete containing micro-nano air bubbles. The studies indicated that MNAB water reduces the workability of fresh concrete and shortens the curing time of cement slurry. Moreover, the authors observed that MNB lowered the hydration temperature.

Table 5 presents the physical properties of lime-cement mortars studied after 28 days of curing in a climatic chamber under the conditions of relative humidity amounting to (90 ± 3)% and air temperature of (20 ± 2) °C.

Oxygen and ozone micro-nano bubble water reduced the specific density of the investigated mortars. The mortars with the carbon-dioxide micro-nano bubble water were characterized by the highest specific density amounting to 2.39–2.44 (g/cm^3^). These mortars also had the highest apparent density and lowest water absorption by weight, 2.7–2.8% lower in comparison to the reference mortars. In turn, substituting mixing water with the ozone and oxygen micro-nano bubble water in 50% and 100%, increased the water absorption by weight. Substituting 50% of mixing water with the oxygen micro-nano bubble water did not indicate a significant difference in water absorption by weight. The 50% O_2_ mortar was characterized by the lowest total porosity. The 100% CO_2_ mortars indicated the highest total porosity; however, this result is comparable to the porosity of the reference sample.

A general decrease in the capillary absorption of mortars with micro-nano bubble water in the range of 0.9–8.5% in relation to the reference mortar. The mortars with mixing water replaced with 100% CO_2_ MNB water, for which the capillary absorption is similar to the reference sample, constitutes an exception.

A decrease in capillary absorption can be attributed to the reduced pore diameter of the lime-cement mortar resulting from the admixture of different nanomaterials. This can be confirmed by the studies conducted by Kyriakou et al. [60]. The authors observed a drop in capillary absorption after 28 days for the lime mortars containing 6% and 9% nano SiO_2_ by 30.7% and 18.5%, respectively, in relation to the reference mortars. However, a reduced increase in the capillary absorption coefficient (mm/min^0.5^) was observed, which was 1.6-fold lower than the mortars with 6% SiO_2_ and halved in comparison to the mortars with 9% nanoaddition. The authors attributed the reduction of capillary absorptivity of the mortars with nanoadditions to the decreased pore dimensions and increased number of gel pores.

Luo et al. [61] observed a reduction in the porosity of lime mortars along with the increased amount of the nano SiO_2_ addition. The authors attributed this phenomenon to the occurrence of pozzolanic reaction between Ca(OH)_2_ and nano SiO_2_. An improvement in the carbonatation process and a reduction in pore diameter were also observed.

The studies by Ergenç et al. [62] on lime-ceramic mortars indicated a reduction in bulk density following the nano Ca(OH)_2_ addition. An increase in open porosity was observed as well. In the hardened mortars, the presence of microcracks filled with precipitated CaCO_3_ was observed, which reduced the diameter of capillary pores, and contributed to the reduction in capillary absorption in relation to other mortars.

Kim et al. [63] observed that submerging the cement mortar in the carbon dioxide micro-nano bubble water multiple times reduces its absorptivity. The authors attributed this process to the faster course of the carbonation reaction in the water with CO_2_ micro-nano bubbles, compared with the carbon dioxide from the atmosphere and the related phenomenon of pores filling with calcium carbonate. A reduction in capillary absorption after 30 min and reduced depth of water infiltration into the concrete following substitution of tap water with the micro-nano bubble water was confirmed by Yahyaei et al. [64]. Moreover, the later studies on various concrete mixes indicated increased compressive and tensile strengths in all investigated hardened concretes despite the lack of significant differences in consistency.

In turn, the studies by Khoshroo et al. [25] indicated a significant influence of MNAB water on the drop of the capillary absorption of concrete both after 30 min and 24 h of the test. Similarly, as in the case of strength, absorptivity decreased along with the increase in the amount of mixing water substitution with MNAB water (30%, 60% and 100% MNB) by 12%, 16% and 20%, respectively, in comparison to the control samples. SEM examination confirmed that the concrete with MNAB is more homogeneous and contains smaller pores than the reference concrete.

Similar conclusions were drawn by Mohsen Zadeh et al. [29]. Substitution of mixing water by micro-nano air bubble water in 50% and 100% caused a reduction in capillary absorption of concrete by 16% and 20%, respectively. Moreover, it was observed that the complete substitution of the mixing water with micro-nano water increased the compressive, tensile and flexural strengths after 28 days by 14%, 18% and 6%, respectively, in relation to the reference concrete. The authors attribute this phenomenon to the increased hydration degree and the properties of bubbles which collide with each other.

The research conducted by Asadollahfardi et al. [28] showed the increase in the compressive strength by 13.2% and tensile strength by 16.4% in relation to the control sample, following the substitution of mixing water with the micro-nano air bubble water. A drop in absorptivity was also observed after 24 h of the 50% or 100% micro-nano bubble water application, reaching 12.5% and 15%, respectively.

**Figure 4:**
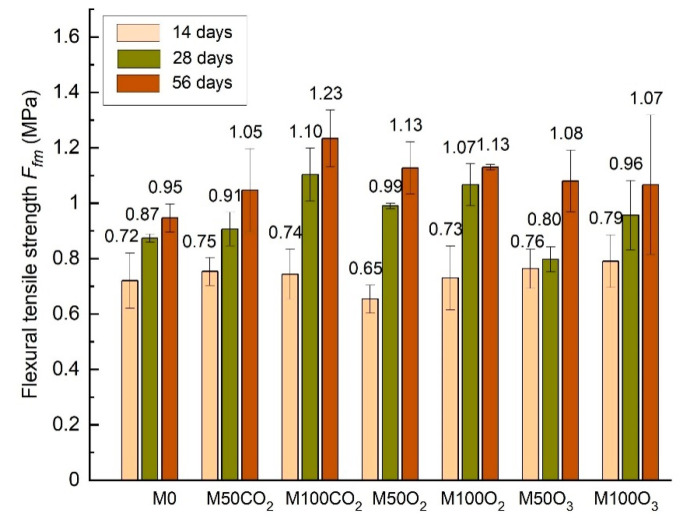
Comparison of the flexural tensile strength of lime-cement mortars with micro-nano bubble water in different periods of time.

**Figure 5:**
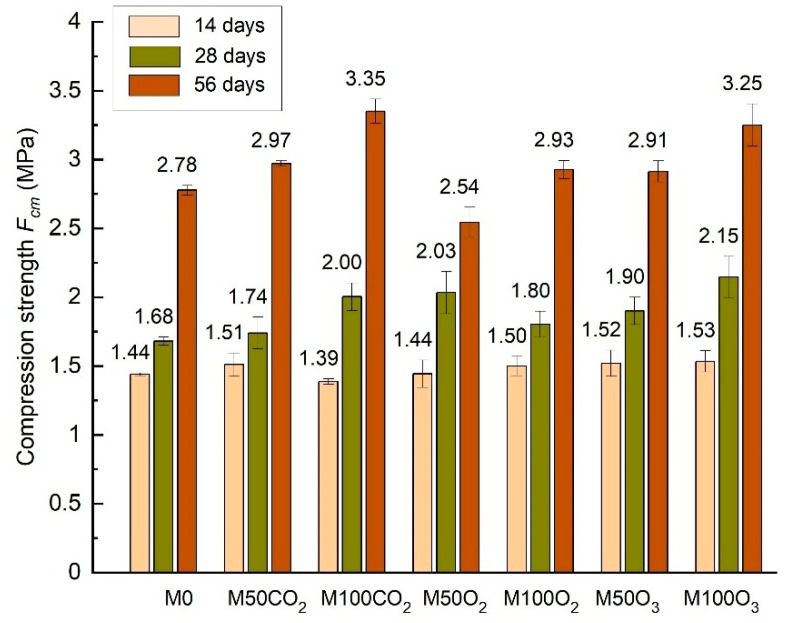
Comparison of the compressive strength of lime-cement mortars with micro-nano bubble water in different periods of time.

Table 6, Figure 4 and Figure 5 present the values of the flexural and compressive strengths determined after 14, 28 and 56 days of curing.

On the basis of the conducted studies, it was stated that substituting mixing water with micro-nano gas bubble water improves the strength of lime-cement mortars. The compressive strength clearly increases along with the quantity of MNB. Nanomaterials, due to their large specific surface, are characterized by increased chemical reactivity, which is suggested by the afore-mentioned literature sources [10,11,12,13,14,15]. The application of micro-nano gas bubble water could accelerate the chemical reaction in the course of curing. The highest compressive strength after 28 days, equal to 2.20 MPa, was reached by the 50% O_2_ mortars. In relation to the reference mortar, this is a 31% increase. In the remaining mortars, the compressive strength increased by 1.2–27.4% compared to the mortar with ordinary water. The examined mortars indicate improved flexural and compressive strengths along with the increasing mixing water substitution. The only exception are the mortars with O_2_, in which the flexural strength dropped by 2.6%, whereas the compressive strength reduced by 18.2%, following the complete substitution of mixing water with micro-nano bubble water—in relation to the 50% substitution. In turn, the highest strength after 56 days was achieved by the mortars with CO_2_, reaching 2.97 and 3.32 (MPa) for the 50% CO_2_ and 100% CO_2_, respectively. Almost all mortars indicated increased flexural strength after 56 days in the range of 8.3–34% and improved compressive strength by 4–19.4%, compared to the reference mortar. Only the 50% O_2_ mortar, which was characterized by the highest compressive strength after 28 days, showed an 8.3% reduction, in relation to the reference mortar.

Literature contains multiple works confirming the influence of nanoadditives on the improvement of the mechanical properties of cement mortars [25,26,27,28,29]. Unfortunately, there are few works on the nanomodifications of lime or lime-cement mortars. The studies by Theodoriduou et al. [65] on the lime mortars modified with nano SiO_2_ and nano TiO_2,_ indicated an improvement of the mechanical properties of all mortars containing nanoadditives. Moreover, the authors observed an increase in the durability of the mortars containing a hydraulic binder with nano SiO_2_ as well as increased resistance to salt crystallization of the mortars with nano TiO_2_.

Kyriakou et al. [60] observed an increased compressive strength of the lime mortars with 9% nano SiO_2_ addition (w/w) after 28 and 90 days by 14.9% and 20%, respectively, in comparison with the reference samples. Moreover, it was observed that the nanomodified mortars exhibited greater hydraulic properties with more hydrated phases and more progressive carbonatation degree. Nanoparticles participate in the binding of lime, precipitating the C-S-H gel, reducing the diameter of pores, and increasing the share of gel pores [66].

Similar conclusions were drawn by Mohsen Zadeh et al. [29]. The micro-nano air bubble water increases the flotation rate of cement particles, raising the share of cement particles in the hydration process. This leads to an increased amount of the C-S-H gel, greater homogeneity of the mixture, and thus, improved mechanical properties of cement composites.

Kim et al. [30] compared the results of the flexural and compressive strengths of the cement mortars prepared with tap water as well as CO_2_ micro-nano bubble water. It was observed that the CO_2_ micro-nano bubble water had higher strength properties in the initial period and yielded comparable results after 28 days, in relation to the reference mortars. The increased strength in the initial period was attributed to the greater amount of CaCO_3_ in the cement matrix, which made the structure of mortars denser.

Khoshroo et al. [25] stated that the concretes with micro-nano air bubble water were characterized by greater compressive and tensile strengths in relation to the control samples. In the case of the compressive strength after 28 and 90 days, 12.5% and 15% increases were noted, respectively. Moreover, an increasing tendency was noted for strength in both periods, along with the increased content of micro-nano bubble water.

Arefi et al. [26] observed that the compressive and tensile strengths were improved owing to MNAB application, by 19% and 16%, respectively.

In turn, Maruyama et al. [67] reported that the micro-nano air bubble water reduces the plastic viscosity of cement slurry and mortars when a polymer admixture is applied and a low w/c ratio is maintained (<0.50). This tendency is more pronounced as the w/c ratio drops. At a w/c ratio equal to 0.5, the authors [67] did not observe similar dependencies. An increased flow time of cement slurry was noted when MNB was applied.

**Figure 6:**
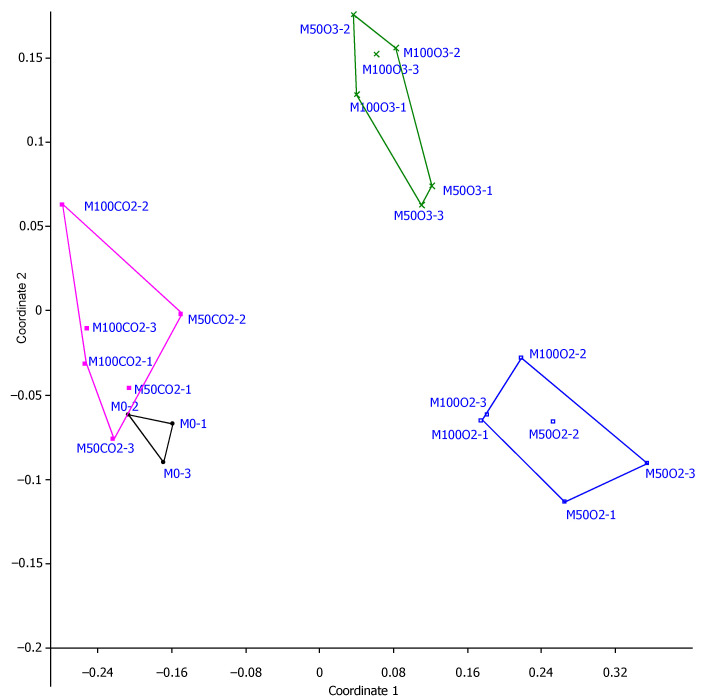
Multidimensional scaling results of adding micro-nano bubble water of the examined gases on mortar properties.

Visualization of the similarity levels was prepared based on the multidimensional scaling method (MDS) for the matrix containing the results of all measurements connected with the properties of the lime-cement mortars along with the 50% and 100% mixing water substitution with O_2_, O_3_ or CO_2_ micro-nano bubble water as well as the reference mortar (see Figure 6).

Figure 6 shows high similarity among the reference mortar samples, which form a tight polygon with short distance between the edges (black lines marked with M0 symbols), compared to the other analyzed mortars modified with varying amounts of MNB water. A polygon visualizing the properties of mortars containing the CO_2_ MNB water is found in close proximity of the afore-mentioned triangle, reflecting the properties and their general variability in the case of the reference mortar. It can be seen that the 50% share of the MNB water contributes to a shorter distance between the visualized points than in the case of the points obtained with 100% share of MNB water. The polygon visualizing the properties of the mortars with CO_2_ MNB also has greater surface and distances between the extreme edges than other polygons, which indicates greater variability of the obtained results and more significant effect of increasing dose on the obtained results of measurements. Another dependence visualized graphically through multidimensional scaling is that the results obtained from the measurements of the mortars with O_2_ addition are more similar (less distant) to the reference mortar compared to the mortars with the O_3_ MNB water. Moreover, relatively short distances between the edges of the polygon reflecting the sum of mortar properties suggests lesser influence (total) on the analyzed parameters of the O_2_ MNB water dose than in the case of the CO_2_ or O_3_ MNB water. In the two-dimensional space, the polygon reflecting the results of measurements related to the mortar with O_3_ was most distant from the triangle visualizing the results of the reference mortar.

**Figure 7:**
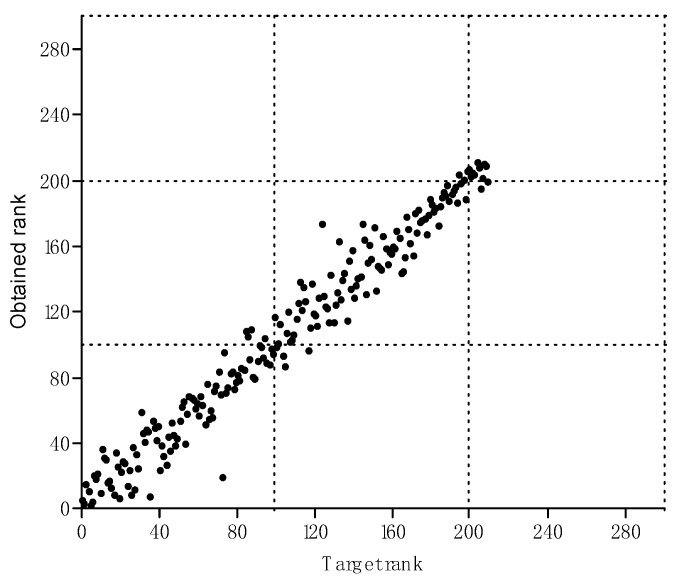
Visualization of the relationships between target rank and obtained rank at STRESS = 0.09 (–).

Another piece of information from the MDS analysis is the low value of STRESS parameter, reaching 0.09, which indicates a good fit of the matrix of the estimated distances to the observed distances. It is additionally supported by a tight point cloud along the plane described by the target rank on the x-axis and the obtained rank on the y-axis (see Figure 7).

## 4. Conclusions

The study investigated the effect of water with micro-nano gas bubbles on the physical and mechanical properties of lime-cement mortars. The mortars with three types of gas (O_2_, O_3_, CO_2_) used as 50% and 100% mixing water substitute and a reference mortar with ordinary tap water were tested. The studies indicated that the micro-nano gas bubble water reduces the workability of lime-cement mortars in all analyzed cases. Since nanomaterials are chemically active, the MNB water affected the cement properties, accelerated the hydration reaction and the early curing process. This phenomenon was observed especially during the compressive and flexural strength tests.

- Along with an increase in the amount of MNB, the compressive strength is markedly proved. The highest strength after 28 days was achieved by the 50% O_2_ mortars, which translates into a 31% improvement in relation to the reference sample. The 50% O_3_ mortar, in which strength dropped by 2.6%, constitutes an exception. Almost all mortars indicated an increase in flexural strength after 56 days, ranging from 8.3% to 34% and increased compressive strength in the range of 4–19.4%, compared to the reference mortar.
- The studies of the physical properties indicated a diversified influence of micro-nano bubbles on the density of mortars. The oxygen and ozone micro-nano bubble water reduced the specific density of the investigated mortars, whereas the water with CO_2_ increased the specific density, which resulted from faster CaCO_3_ formation in the mortar. The CO_2_ micro-nano bubble water increased the bulk density of the considered mortars by 0.9% and 1.5% in the case of the 50% CO_2_ and 100% CO_2_, respectively. These mortars were also characterized by a comparable porosity to that of the reference mortars, reaching the highest porosity values out of all mortars.
- A general reduction in capillary absorption of the mortars with micro-nano bubble water was observed in the range of 0.9–8.5% in relation to the reference mortar. This is caused by a reduced size of capillary pores resulting from the application of MNB water, which is confirmed by the studies concerning the water absorption by weight of the mortars with ozone and oxygen micro-nano bubbles, which reduced the water absorption of the investigated mortars by 2.7–2.8% in relation to the reference mortars.
- The analysis based on the MDS method showed that taking into account the results of all analyzed parameters simultaneously, it is possible to clearly distinguish the mortars prepared with the addition of the water containing micro-nano bubbles of various gases as well as reference mortars. The changes in the properties of the mortar are most clearly visible with the increased proportion of water with the CO_2_ micro-nano bubbles as mixing water.

The ozone, oxygen, and carbon dioxide micro-nano bubble water proved to be a desirable substitute of tap water, ensuring a simultaneous improvement of the physico-mechanical properties of lime-cement mortars. The application of MNB is technologically possible in almost each case and does not require complicated procedures, compared to other nanoadditives used in the construction industry. The works on the prospective application of the water with micro-nano bubbles of various gases in the process of improving the strength of cement composites, as well as the investigations connected with the chemical analysis and determination of the mechanisms occurring in the internal structure of the studied lime-cement mortars are currently underway.

## Figures and Tables

**Table 1:** Composition of lime-cement mortars.

Components	Unit	Reference	50% CO_2_	100% CO_2_	50% O_2_	100% O_2_	50% O_3_	100% O_3_
Hydrated lime	(kg/m^3^)	518.1	518.1	518.1	518.1	518.1	518.1	518.1
White cement 52.5R	(kg/m^3^)	172.7	172.7	172.7	172.7	172.7	172.7	172.7
Natural quartz sand 0–2.0 mm	(kg/m^3^)	518.1	518.1	518.1	518.1	518.1	518.1	518.1
Water	(L/m^3^)	518.1	259.05	–	259.05	–	259.05	–
Micro-nano water with CO_2_	(L/m^3^)	–	259.05	518.1	–	–	–	–
Micro-nano water with O_2_	(L/m^3^)	–	–	–	259.05	518.1		
Micro-nano water with O_3_	(L/m^3^)	–	–	–	–	–	259.05	518.1

**Table 2:** Parameters of hydrated lime [36].

Parameter		Unit	Value
Activated lime content		(%, weight)	≥80.0
Volume constant		(%, mm)	≤4.0
Infiltration depth		(mm)	>10 and <25
Air content		(%, volume)	≤5.0
Degree of grinding	Remains on the 0.09 mm sieve	(%)	≤0.2
	Remains on the 0.20 mm sieve		≤0.2
Free water content		(%)	≤0.2
Bulk density		(kg/dm^3^)	0.51
Chemical composition	CaO + MgO	(%)	≥93.0
	MgO		≤1.2
	CO_2_		≤3.5
	SO_3_		≤1.0

**Table 3:** Parameters of white Portland cement CEM I 52.5 R [37].

Parameter		Unit	Value
Initial setting time		(min)	120
Sulfate resistance		(%)	C_3_A ≤ 4–5
Alkali content		(%)	≤0.3
Specific surface		(m^2^/kg)	400
Chemical composition of cement	C_3_S	(%)	77
	C_2_S		16
	C_3_A		5
	C_4_AF		1
	others		1
Compressive strength after	1 day	(MPa)	23
	2 days		42
	7 days		60
	28 days		72

**Table 4:** Consistency of fresh lime-cement mortars.

	Reference	50% CO_2_	100% CO_2_	50% O_2_	100% O_2_	50% O_3_	100% O_3_
Flow diameter (cm)	20.0	17.8	16.4	18.3	16.8	19.0	17.5

**Table 5:** The physical parameters of the tested lime-cement mortars after 28 days of curing in a climatic chamber (humidity: (90 ± 3)%; air temperature: (20 ± 2) °C).

	Specific Density	Bulk Density	Water Absorptivity	Total Porosity	Water Absorption Coefficient
	(g/cm^3^)	(kg/m^3^)	(%)	(%)	(kg/m^2^)
Reference	2.38	1287	35.89	45.92	31.65
50% CO_2_	2.39	1299	34.87	45.48	31.36
100% CO_2_	2.44	1306	34.93	46.01	31.73
50% O_2_	2.14	1292	35.86	39.76	29.64
100% O_2_	2.16	1281	36.56	40.79	30.16
50% O_3_	2.26	1288	36.37	43.11	28.95
100% O_3_	2.24	1255	36.87	43.94	29.16

**Table 6:** The flexural tensile and compression strengths of mortars after 14, 28 and 56 days.

	*F_fm_*_,14_	*F_cm_*_,14_	*F_fm_*_,28_	*F_cm_*_,28_	*F_fm_*_,56_	*F_cm_*_,56_
	(MPa)	(MPa)	(MPa)	(MPa)	(MPa)	(MPa)
Reference	0.72	1.44	0.87	1.68	0.94	2.78
50% CO_2_	0.76	1.50	0.90	1.70	1.04	2.97
100% CO_2_	0.78	1.38	1.10	2.01	1.26	3.32
50% O_2_	0.66	1.46	0.99	2.20	1.16	2.55
100% O_2_	0.72	1.50	1.05	1.80	1.13	2.91
50% O_3_	0.75	1.50	0.80	1.90	1.06	2.89
100% O_3_	0.78	1.55	0.90	2.14	1.10	3.21

## Data Availability

Obtained data are presented in paper.
